# Precious1GPT: multimodal transformer-based transfer learning for aging clock development and feature importance analysis for aging and age-related disease target discovery

**DOI:** 10.18632/aging.204788

**Published:** 2023-06-13

**Authors:** Anatoly Urban, Denis Sidorenko, Diana Zagirova, Ekaterina Kozlova, Aleksandr Kalashnikov, Stefan Pushkov, Vladimir Naumov, Viktoria Sarkisova, Geoffrey Ho Duen Leung, Hoi Wing Leung, Frank W. Pun, Ivan V. Ozerov, Alex Aliper, Feng Ren, Alex Zhavoronkov

**Affiliations:** 1 Insilico Medicine, Pak Shek Kok, New Territories, Hong Kong; 2 Insilico Medicine, Masdar City, United Arab Emirates; 3 Insilico Medicine, Shanghai, China

**Keywords:** transformers, deep learning, therapeutic target discovery, aging biomarkers, human aging

## Abstract

Aging is a complex and multifactorial process that increases the risk of various age-related diseases and there are many aging clocks that can accurately predict chronological age, mortality, and health status. These clocks are disconnected and are rarely fit for therapeutic target discovery. In this study, we propose a novel approach to multimodal aging clock we call Precious1GPT utilizing methylation and transcriptomic data for interpretable age prediction and target discovery developed using a transformer-based model and transfer learning for case-control classification. While the accuracy of the multimodal transformer is lower within each individual data type compared to the state of art specialized aging clocks based on methylation or transcriptomic data separately it may have higher practical utility for target discovery. This method provides the ability to discover novel therapeutic targets that hypothetically may be able to reverse or accelerate biological age providing a pathway for therapeutic drug discovery and validation using the aging clock. In addition, we provide a list of promising targets annotated using the PandaOmics industrial target discovery platform.

## INTRODUCTION

Aging is a complex, multifactorial process that results from a multitude of interacting biological mechanisms occurring at different levels within an organism [1]. The development of accurate, physiologically meaningful biomarkers of aging is crucial for assessing the efficacy of potential anti-aging therapies and advancing the field of aging research [2, 3]. Deep neural networks (DNNs) have demonstrated remarkable success in various applications, including biomedical research [4, 5]. Population-specific aging clocks have been developed using large datasets from diverse ethnic groups, enabling more accurate predictions of chronological age and biological age, as well as assessment of all-cause mortality [6]. Moreover, artificial intelligence (AI)-driven platforms, such as PandaOmics, have facilitated the identification and prioritization of novel aging-associated targets for drug discovery and repurposing [7]. Recent studies have also demonstrated the value of AI in advancing longevity research by harnessing the power of next-generation sequencing data and omics technologies [8].

Insilico Medicine has been at the forefront of using generative AI in biology since 2016 [9–11]. Their research has led to the development of generative biology approaches that utilize generative systems to generate synthetic biological data, including their first successful demonstration taking place at the National Institute of Aging [12]. In addition to target discovery, Insilico has also developed capabilities in generative chemistry [4, 13–15]. These approaches have been successfully applied to various diseases and aging and have shown potential in identifying novel compounds and accelerating drug development [16–18]. As the aging population continues to grow, there is an urgent need for new therapeutic targets to delay and treat age-related diseases. Therefore, the application of generative biology approaches in exploring the complex interplay between aging and diseases holds great promise for identifying potential novel targets and accelerating drug development efforts.

Deep aging clocks have been developed for various applications in pharmaceutical research and development. For example, DeepMAge, a methylation aging clock developed using deep learning, shows remarkable accuracy and biological relevance in predicting human age and identifying health-related conditions [19]. Moreover, deep aging clocks could potentially be used for target identification, drug discovery, data quality control, and synthetic patient data generation [3]. Additionally, the use of AI to comprehend the intricate interplay between the microenvironment within the human body and the external environment has shown promise in revealing the role of external factors in aging [8]. The integration of deep learning techniques with genomics and other omics data has enabled comprehensive comparisons of DNA repair transcriptomes in species with extreme lifespan differences, shedding light on the potential role of DNA repair as a longevity assurance system [20].

Despite the progress made in developing deep aging clocks and AI-based biomarkers, there are still several challenges and opportunities for improvement in the field of biohorology [16]. The development of deep learning, DNNs, and generative approaches is expected to significantly advance the field, leading to more accurate and robust aging biomarkers [16]. Furthermore, *in silico* methods for screening and ranking potential geroprotective candidates, based on their ability to regulate age-related changes in signaling pathway clouds, hold promise for accelerating the discovery of effective interventions and reducing the time and cost of pre-clinical work and clinical trials [21]. For instance, GeroScope could predict novel geroprotectors from existing human gene expression data by mapping expression differences between young and old subjects to age-related signaling pathways and ranking known substances (potential geroprotector candidates) based on their likelihood to target differential pathways and mimic the young signalome [22]. Similarly, the human gut microbiome has been shown to have a strong association with host age, and deep learning-based models have been developed to predict host age based on gut microflora taxonomic profiles, further providing insights into potential aging biomarkers [17].

To identify aging biomarkers associated with age-related diseases, in the present work, we combined the ability of aging clocks to predict biological age and thus grasp molecular changes accompanied by senescence and our target ID approach to establish genes that are related to the development of diseases. This provides us with a novel perspective on uncovering the molecular mechanisms of diseases in the context of aging, allowing us to identify promising strategies to delay and treat age-related diseases.

## RESULTS

### Performance of the transformer-based multimodal aging clock

**Figure 1:**
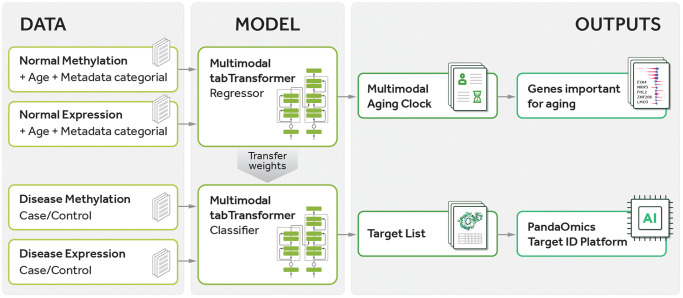
**Pipeline of the current study.** The pipeline involves training a multimodal transformer-based regressor on normal sample data to predict age, followed by transferring the learned weights to a transformer-based classifier for distinguishing between case and control samples. Gene prioritization is then performed using feature importance values obtained from the regressor to rank genes according to their relevance to aging and using importance values from the classifier to rank genes according to their relevance to both aging and disease. Finally, the gene lists are analyzed using the PandaOmics TargetID Platform.

In the current study, we have developed a comprehensive pipeline, as illustrated in Figure 1. The pipeline consists of several key steps, including training a multimodal transformer-based regressor on normal sample data for age prediction and subsequently using the learned weights to fine-tune a transformer-based classifier for distinguishing between case and control samples. Next, we perform gene prioritization by employing the feature importance values obtained from the regressor to rank genes based on their relevance to aging and utilizing the importance values from the classifier to rank genes in terms of their relevance to both aging and disease. Finally, we analyze the resulting gene lists using the PandaOmics TargetID Platform to gain insights into potential targets for age-related diseases. In this study, we implemented a transformer-based architecture to accommodate both numerical and categorical data as input for our predictive model. This strategy, which we call Precious1GPT, enables the construction of multimodal classifiers and regressors that can effectively process diverse data types, such as RNA-seq expression data and epigenomics methylation data taking into account data type and tissue type. Consequently, our model demonstrates proficiency in age prediction and case-control classification, showcasing its versatility in handling multifaceted inputs.

We employed Optuna [23], a hyperparameter optimizer, to optimize the parameters for each model. We optimized L1 and L2 regularization, the activation function, dropout value, batch size, the number of neurons in hidden layers, and the gradient update used. The final regression metrics for the optimized models are shown in Table 1, calculated for the epigenetic and expression sub-datasets and for the whole dataset, respectively. The metrics were calculated by splitting the data to train (80%) and test (20%) set with stratification by sample tissue. For five-fold cross-validation stratified by tissue and data modality, results are shown in Supplementary Table 1. Metrics for individual tissues are shown in Supplementary Table 2. The methylation data subset, expression data subset, and all the test data combined were also calculated for each metric. Learning curves depicting MAE during training are shown in Supplementary Figure 1.

**Table 1:** Multimodal transformer-based regressor metrics were evaluated on the hold-out test dataset (20% of all data).

**Metric**	**Methylation data**	**Expression data**	**Combined**
MAE	4.227	6.287	5.622
RMSE	6.129	8.155	7.560
R^2^	0.934	0.584	0.807
MdAE	2.880	5.098	4.336
Number of samples in test set	4,019	2,730	6,749

### Important genes for age prediction

SHapley Additive exPlanations (SHAP) values [24] are a technique for explaining the output of machine learning models. From the regressor model, we obtained a list of features ranked by their SHAP values representing their importance for age prediction (Supplementary Table 3). Pathway enrichment analysis was subsequently performed for the top-100 genes ranked based on the SHAP values, which showed that these genes are implicated in multiple pathways associated with aging and age-related diseases (Table 2).

**Table 2:** Reactome pathway analysis results for the top-100 genes selected based on the SHAP values.

**Pathway**	***P*-value**	**Odds ratio**	**Combined score**
tRNA Processing in Mitochondrion R-HSA-6785470	0.036	33.99	112.78
Amino Acid Transport Across Plasma Membrane R-HSA-352230	0.002	13.77	86.96
Suppression Of Apoptosis R-HSA-9635465	0.043	27.19	85.36
Vasopressin-like Receptors R-HSA-388479	0.043	27.19	85.36
Highly Sodium Permeable Postsynaptic Acetylcholine Nicotinic Receptors R-HSA-629587	0.050	22.66	67.72
Cytosolic Sulfonation of Small Molecules R-HSA-156584	0.011	13.68	61.37

### Identification of potential targets for age-related diseases through feature importance analysis

Utilizing the feature importance analysis based on SHAP values, we generated a list of genes associated with aging (Supplementary Table 3). We then compared these genes with known drug targets in our in-house database to identify potential therapeutic interventions for 4 selected age-related diseases, namely idiopathic pulmonary fibrosis (Supplementary Table 4), chronic obstructive pulmonary disease (COPD) (Supplementary Table 5), Parkinson’s disease (PD) (Supplementary Table 6) and heart failure (Supplementary Table 7). Evaluation metrics for case-control classifiers are shown in Supplementary Table 8.

We adopted a transfer learning approach to identify genes involved in disease development in the context of aging. We first trained a DL-model as a regressor to predict age using an age dataset. Subsequently, we fine-tuned the model by re-training it as a case-control classifier while keeping the previously learned weights frozen, with the exception of the last layer. The SHAP values generated from this analysis were then used to determine the relative importance of molecular features in driving disease development in the context of aging. This allowed us to identify specific genes that are involved in disease development in the context of aging and determine their relative importance. To establish a baseline, we trained the same classifiers on the complete feature set. For a number of diseases, we observed a slight but significant increase in classification metrics (Supplementary Table 8). These results indicated that the last layer of the neural network, which was trained to predict biological age, contains sufficient information to differentiate between case and control.

**Figure 2:**
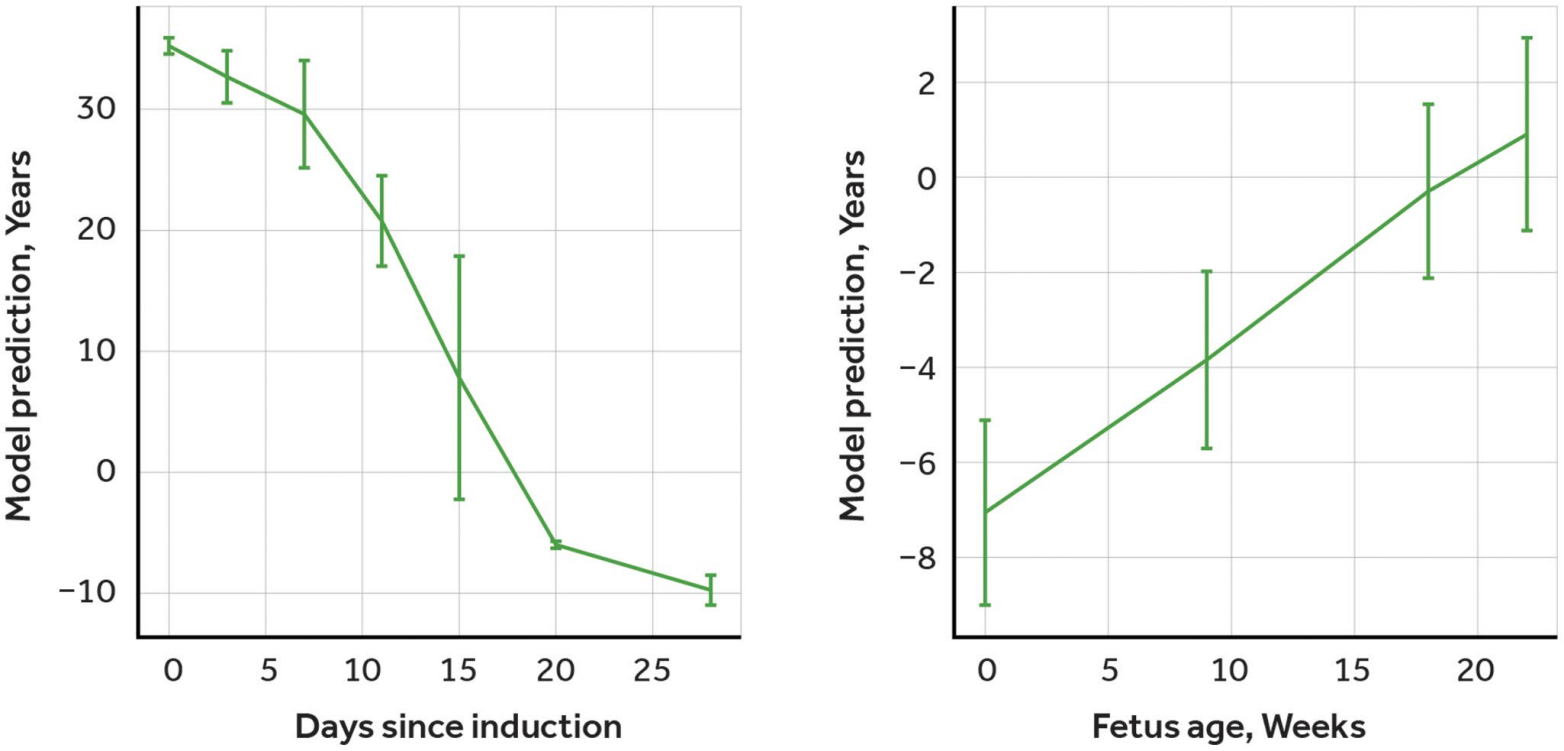
**Validation of age-predictor model using induced pluripotent stem cells (iPSC) and fetal tissues methylation data.** Left: Predictions of the multimodal transformer for iPSC induction dataset, days after transfection with reprogramming factors. Right: Predictions of the multimodal transformer for embryonic tissue dataset, weeks after last menstruation, averaged across tissues.

To validate the performance of our model and to establish its ability to accurately estimate age based on methylation data, we acquired two methylation datasets from the Gene Expression Omnibus (GEO) repository - one on cells that were reprogrammed to induced pluripotent stem cells (iPSC) (dataset GSE54848) and the other on fibroblasts of the developing fetuses (dataset GSE76641). The selection of the independent dataset on the developing cells, along with reverse aging cells data, allows us to provide another level of validation evidence confirming the potency of the trained model. These datasets were processed using the same methods as our primary dataset on age-related methylation, and we used the model to predict age. In order to increase the robustness of our validation, we used the same processing methods for both the iPSC and fetus datasets as we had for our primary dataset. This ensured consistency and minimized the possibility of any discrepancies or variations in our results due to differences in processing methods. The results were consistent with expectations, as iPSCs become younger during induction (Figure 2, Left) and fetal tissue becomes older during development (Figure 2, Right).

### Manual analysis of the resulting targets

**Figure 3:**
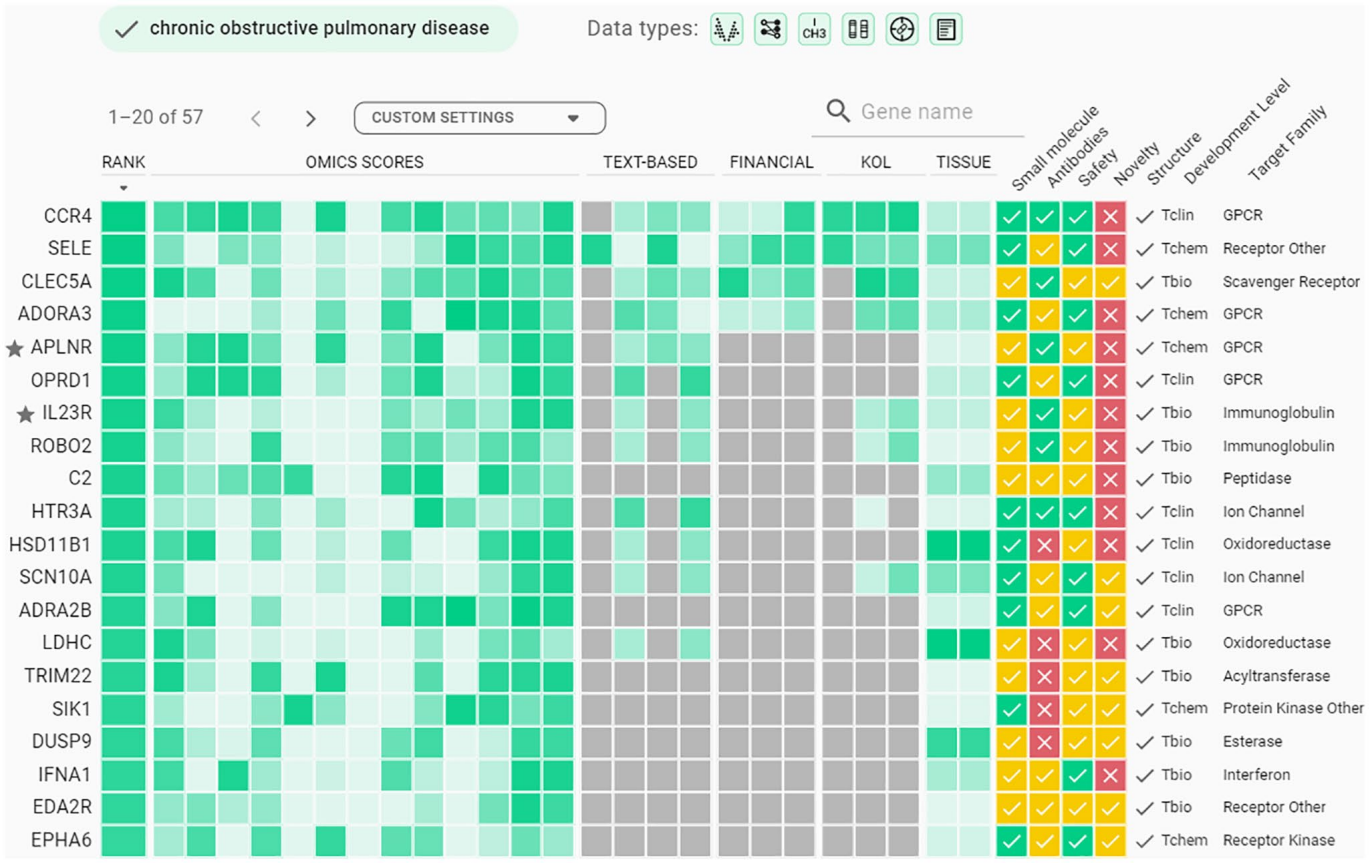
**Example of Target ID output for chronic obstructive pulmonary disease.** Top-200 genes from expression classifiers were applied as a gene list in PandaOmics corresponding project for COPD, and a filter for small molecules was applied to identify druggable targets. Twenty genes highly ranked by PandaOmics are shown.

Utilizing a transfer learning approach, we have built aging-aware case-control classifiers and extracted feature importance values from them. The lists of top-200 genes ranked by expression classifiers were retrieved (Supplementary Tables 4–7) and considered as a starting point for further target identification and prioritization techniques offered by the AI-powered PandaOmics platform to propose a list of promising novel targets for age-related diseases. According to PandaOmics TargetID platform, APLNR was ranked top-20 for all 4 diseases, while IL23R was ranked top-20 for COPD, PD, and heart failure (Figure 3, Supplementary Figures 2–4). APLNR and IL23R were therefore selected as the most promising targets for treating multiple age-related diseases. In general, APLNR, a receptor for Apelin and Elabela peptide ligands, is involved in regulating several important physiological processes, including cardiovascular function, fluid balance, and metabolism. IL23R is a receptor for the pro-inflammatory cytokine IL-23 and is associated with chronic inflammation, which is considered to be one of the hallmarks of aging [25]. Accumulating evidence demonstrated that the effectiveness of our approach in addressing various age-related diseases, as documented in existing literature, provides further validation for our approach. Therefore, our unique approach with the amplification of PandaOmics allows us to identify various potential targets associated with essential aging-driven tissue dysfunction, which may be useful for the delay and treatment of multiple age-related diseases.

## DISCUSSION

The development of “aging clocks,” based on machine learning models that predict age based on biological data, has become a major milestone in aging research. Nevertheless, such an approach has severe limitations, such as a lack of ability to explain biological processes accompanied by aging and, thus, the ability to propose therapeutic interventions to compensate for age-related deterioration [26]. Additionally, aging clocks can be used to monitor the effectiveness of interventions and therapies designed to target age-related diseases [27]. For example, if an intervention is able to slow down the aging process, as measured by the aging clock, it may be more likely to be effective in delaying or treating age-related diseases. This can be done by comparing the aging clock values of an individual before and after the intervention and measuring the change in the aging clock value, which may indicate the effectiveness of the intervention. The development of Precious1GPT, a multimodal aging clock using a transformer-based model and transfer learning for case-control classification, as well as the identification of potential therapeutic targets for age-related diseases through feature importance analysis, has demonstrated the potential of our approach in deciphering the molecular mechanisms of aging. The transformer-based model allowed for the integration of multi-omics data and improved the accuracy of the aging clock, while the transfer learning approach facilitated the identification of disease-related genes in the context of aging. However, our study has several limitations, including the reliance on publicly available datasets, which may contain noisy and low-quality data. Future research should focus on validating the identified targets using experimental methods and exploring the potential of new drug targets.

Several aging clocks have been proposed in the literature, each with its own strengths and limitations. Some of the most prominent aging clocks include the Epigenetic Clock, DNAm PhenoAge, and the transcriptomic-based Aging.AI clock [28–31]. These clocks utilize various molecular markers, such as DNA methylation or gene expression patterns, to predict an individual’s chronological age. Our proposed multimodal aging clock uses a transformer-based model and transfers learning to integrate diverse data sources, including epigenetic and transcriptomic data, and to predict age with high accuracy. When compared to existing aging clocks, our approach demonstrates several advantages. First, the multimodal nature of our approach enables the integration of different omics data types, leading to a more comprehensive and accurate assessment of an individual’s biological age. By incorporating multiple data types, our aging clock can capture a wider range of molecular changes associated with aging, leading to a more reliable and informative model. Second, the use of transformer-based deep learning models allows our approach to capturing complex relationships between features, which can lead to improved age prediction accuracy. In contrast, traditional aging clocks like the Epigenetic Clock and DNAm PhenoAge rely on linear regression models, which may not be able to fully capture the complexity of age-related molecular changes. Third, our approach employs transfer learning for case-control classification, enabling the identification of potential targets for age-related diseases. This aspect of our method offers a significant advantage of Precious1GPT over existing aging clocks, as it not only allows for accurate age prediction but also contributes to the discovery of novel therapeutic targets for age-related diseases.

Unexpectedly, our model could not identify the genes which play key roles in known age-related pathways (SIRT, mTOR, and AMPK) as important genes (i.e., top-200) for age prediction. However, such a phenomenon was also observed in published DL age prediction models [32]. To further test if the most important genes (i.e., top-100) share any biological features, we performed pathway enrichment analysis which revealed that the list of identified genes is significantly enriched in the following pathways that are associated with the aging process:

1. tRNA Processing in Mitochondrion (R-HSA-6785470): This pathway is involved in the processing of transfer RNAs (tRNAs) within the mitochondria, which is essential for proper mitochondrial protein synthesis and overall mitochondrial function. Mitochondrial dysfunction has been implicated in the aging process and age-related diseases, such as neurodegenerative disorders and metabolic syndromes [33].
2. Amino Acid Transport Across Plasma Membrane (R-HSA-352230): This pathway describes the transport of amino acids across the plasma membrane, a crucial process for maintaining cellular homeostasis and protein synthesis. Dysregulation of amino acid transport may lead to imbalances in protein synthesis and degradation, which could contribute to cellular senescence, a hallmark of aging [25].
3. Suppression of Apoptosis (R-HSA-9635465): This pathway is involved in the regulation of apoptosis, a crucial cellular process that controls cell death and tissue homeostasis. Dysregulation of apoptosis has been linked to aging and age-related diseases such as cancer, neurodegenerative disorders, and cardiovascular diseases [34].
4. Vasopressin-like Receptors (R-HSA-388479): This pathway focuses on the signaling of vasopressin-like receptors, which play a role in water homeostasis, blood pressure regulation, and stress response. Alterations in these processes have been associated with age-related physiological changes, such as decreased stress resilience and increased risk of hypertension [35].
5. Highly Sodium Permeable Postsynaptic Acetylcholine Nicotinic Receptors (R-HSA-629587): This pathway deals with the function of acetylcholine nicotinic receptors, which are involved in neurotransmission and neuromuscular function. Impairment of neurotransmission and synaptic function has been implicated in aging and age-related neurodegenerative disorders, such as Alzheimer's and Parkinson’s diseases [36].
6. Cytosolic Sulfonation of Small Molecules (R-HSA-156584): This pathway describes the process of cytosolic sulfonation, a phase II detoxification reaction that helps to maintain cellular redox homeostasis and protects cells from oxidative stress. Oxidative stress has been widely recognized as a major contributor to the aging process and the development of age-related diseases.

The transfer learning approach utilized in this study enabled the construction of aging-centered case-control classifiers. These models were used to obtain lists of genes ranked by both their association with aging and diseases. Fibrotic disease, inflammatory disease, neurological disease, and cardiovascular disease are common disease classes in humans. To represent these categories, we have selected different age-related diseases, including idiopathic pulmonary fibrosis, COPD, PD, and heart failure, for each disease class. With the application of PandaOmics, APLNR, and IL23R are identified as the most potential aging targets for delaying and treating multiple age-related diseases. APLNR was in top-20 predictions for all four selected diseases. A declining Apelin/APLNR signaling promotes aging, whereas its restoration extended healthspan [37], and endogenous Apelin is protective against age-related loss of retinal ganglion cells in mice [38], further revealing its critical role in regulating aging. While the expression of both Apelin and APLNR decreases with increasing age [37], agonism of apelin receptors produces beneficial effects in fibrotic, cardiovascular, and cognitive disorders [39–41]. Taken together, targeting Apelin-APLNR signaling represents a very promising approach for the treatment of multiple age-related complications. Another potential multi-disease target that was in top-20 predictions for COPD, PD, and heart failure is IL23R, a receptor for IL-23 pro-inflammatory cytokine. Upregulation of the p19 subunit expression and IL-23 protein production in dendritic cells was observed in aged mice and may represent a potential mechanism for inadequate inflammatory responses in aging [42]. In the COPD murine model, IL-23^−/−^ mice developed significantly lower static compliance values and decreased emphysematous changes in the lung tissue compared to WT mice [43]. Though the role of IL-23 is understudied in PD, neuroinflammation is a typical pathological feature of many neurodegenerative diseases, while emerging evidence indicates that sustained activation of microglia and astrocytes is central to dopaminergic degeneration in PD [44]. IL-23 can also enhance age-associated inflammation in Alzheimer’s disease [45], likely to cause the accumulation of cellular damage and compromise the body’s ability to repair itself. Local production of IL-23 in the Central Nervous System has been demonstrated for astrocytes and infiltrating macrophages under inflammatory conditions [46]. Altogether, agonizing Apelin/APLNR signaling and antagonizing IL23/IL23R axis may serve as potential therapeutic strategies for delaying and treating multiple age-related complications. These findings provide insights into potential targets for the delay and treatment of age-related diseases and demonstrate the utility of the transfer learning approach in identifying important genes associated with age-related dysfunction.

As there is a huge bet on AI and transformer applications in biomedicine, we expect future studies to focus on developing further the approach proposed in this paper, including possible integration of larger proprietary disease-specific datasets and validation of the identified targets in the wet lab setting. Moreover, exploring the potential of new drug targets and optimizing our model’s performance will be crucial for advancing our understanding of the molecular mechanisms of aging and developing reliable interventions for age-related diseases. Ultimately, there is a great hope that Precious1GPT and its continued development and refinement will contribute significantly to the improvement of human health and longevity.

## MATERIALS AND METHODS

### Data sources for multimodal aging clock development

To train our models, we used several datasets, including publicly available and in-house-built ones. For training age prediction models based on the epigenetic status of tissue, we employed 450 k Illumina Methylation array data from EWAS Data Hub [47] (number of samples = 8,374).

For building age prediction models based on the transcriptomics status of tissue, we employed RNA-Sequencing data from the GTEx project [48] (number of samples = 12,453). We trained our models in a tissue-agnostic fashion. For methylation, data distribution of samples across ages is shown in Supplementary Figure 5A and across tissues in Supplementary Figure 5B. For expression data, distribution of samples across ages is shown in Supplementary Figure 6A, across tissues in Supplementary Figure 6B.

For assessing prediction results and predicting disease targets using age-pretrained models, we used custom datasets from the PandaOmics software [49] for 4 selected age-related diseases: idiopathic pulmonary fibrosis, COPD, PD, and heart failure from where we have obtained samples annotated as carrying disease (case samples) and healthy ones (control samples).

To evaluate possible interventions to prevent senescence development, we have constructed datasets containing only features corresponding to genes for which approved drugs exist. For this, we have used an in-house constructed database of approved drugs and their targets based on the information from [50].

As input features for our age prediction models were either beta values averaged across CpG probes annotated as TS200 region from Illumina 450k Methylation Array for epigenomic data or TPM values for protein coding (genes) for expression datasets accordingly.

For the DNA methylation data, we obtained the ß-values from the CNCB data hub, where the raw data were obtained using the GMQN package developed by the CNCB [47].

In our study, we have opted for the TSS200 region as the most interpretable for age prediction. Following the aggregation of corresponding beta-values, we are left with approximately 14,000 features representing average methylation of proximal promoter regions.

This choice of region is based on its potential to offer a more accurate and comprehensive assessment of age-related changes in methylation patterns. The TSS200 region, situated within 200 base pairs upstream of the transcription start site, is known to play a crucial role in gene regulation [51]. As such, it is expected to exhibit significant age-related changes in methylation patterns, providing a robust basis for predicting biological age.

To further enhance the interpretability of our age prediction model, we utilized machine learning techniques to identify and select the most informative features from the 14,000-feature dataset. This allowed us to refine our model, ensuring that it captures the most relevant age-related methylation changes in the TSS200 region. In addition, the selected features can potentially shed light on the molecular mechanisms underlying aging, as well as the development of age-related diseases.

By focusing on the TSS200 region, we aim to not only improve the accuracy of our aging clock but also gain a deeper understanding of the complex relationship between DNA methylation, aging, and age-dependent diseases. This knowledge can then be used to develop targeted therapeutic interventions aimed at mitigating the impact of age-related diseases and improving overall health and quality of life in aging populations.

For gene expression models, we have used TPM values which were back-corrected using ComBat [52] and quantile-normalized using qnorm [53]. For performance evaluations, we employed a shuffle split stratified by sample tissue, leaving 20% of all data for the test set.

### Transformer-based model for multimodal aging clock

Given the abundance of omics data available for various experimental conditions and the distinct challenges of predicting one omic data type from another, we propose the development of a transformer-based model to estimate sample age across different sample types. This multi-tissue, multi-omics transformer-based age prediction model aims to harness the power of deep learning to effectively integrate diverse data sources and improve age prediction accuracy.

Transformers have demonstrated remarkable success in various applications, particularly in natural language processing tasks. Their capacity to model long-range dependencies and capture complex relationships among features makes them well-suited for multi-omics data integration. By leveraging the transformer architecture, our proposed model is able to identify and exploit relevant information from different omics data types, such as genomics, transcriptomics, proteomics, and metabolomics, as well as different tissue types.

By developing a multi-tissue, multi-omics transformer-based age prediction model, we hope to enhance the accuracy and generalizability of aging clocks, ultimately contributing to a better understanding of the aging process and facilitating the development of targeted therapies for age-related diseases.

More formally about the transformer model. Let *X* be the input matrix of size (*N*, *D*) containing epigenetic (methylation) and expression data, and *Z* be the input matrix of size (*N*, *M*) containing categorical data. Let *Y* be the output matrix of size (*N*, 1) containing the predicted age values. Since we used TabTransformer (LINK), we represented the model as a function *F* that takes *X* and *Z* as input and returns Y as output: *Y* = *F* (*X*, *Z*). The model consists of multiple linear and attention layers, each of which applies a set of learnable parameters to the input and produces an output. We represent each layer as a function *G* that takes an input matrix *A* and a set of learnable parameters *W* and *b*, and produces an output matrix:


B:B=G(A,W,b)=ReLU(AW+b)B=G(A,W,b)=ReLU(AW+b)

The TabTransformer [54] model consists of multiple layers, including self-attention layers and feedforward layers. Let A be the output of the previous layer, and let W_*q*_, W_*k*_, and W_*v*_ be learnable weight matrices of size (*D*, d_*k*_). The self-attention layer computes the attention matrix *A’* as follows:


$$A^{\prime }=soft\max\left(\frac{AW_{q}{\left(W_{k}\right)}^{T}}{\sqrt{d_{k}}}\right)W_{v}O=A^{\prime }W_{v}$$

where W_*q*_, W_*k*_, and W_*v*_ are weight matrices of size (*D*, d_*k*_). The feedforward layer computes the output matrix *H* as follows:


$$H=G\left(O,W_{1},b_{1}\right)=ReLU\left(OW_{1}+b_{1}\right)$$

where *W*_1_ is a weight matrix of size (*h*, *d*_h_), and *b*_1_ is a bias vector of size (1, *d*_h_). We repeat the self-attention and feedforward layers multiple times to create a deep TabTransformer model. Next, we apply a feedforward layer with weight matrix *W*_1_ and bias *b*_1_ to the output of the self-attention layer: *H* = *ReLU* (*OW*_1_ + *b*_1_). Finally, we apply a linear layer with weight matrix *W*_2_ and bias *b*_2_ to the output of the last feedforward layer to produce the final output matrix *Y*:


$$Y=HW_{2}+b_{2}$$

To train the TabTransformer model, we used mean squared error (MSE) loss that measures the difference between the predicted age values and the true age values. Let $\hat{Y}$ be the predicted age values and *Y_true_* be the true age values:


$$L=\frac{1}{N}\sum_{i=1}^{N}{\text{(}{\hat{Y}}_{l}-Y_{true,i}\text{)}}^{2}$$

Overall, the experiment involves training a TabTransformer model on a dataset containing epigenetic (methylation) and expression data, as well as categorical data (tissue and dataset type), to predict age values. The model consists of multiple layers, including self-attention and feedforward layers, and is trained using a loss function such as MSE.

In the present study, we utilized PyTorch Tabular [55], which is built on top of PyTorch, for all the work with the transformer. PyTorch Tabular provides a highly optimized and efficient way of handling tabular data with PyTorch. We used PyTorch Tabular’s various functions and modules to preprocess the input data, construct the transformer architecture, and train it on the data.

In the model, all the hyperparameters, such as learning rate, dropout rate, number of hidden layers, and activation functions, were determined through the use of Optuna [23], a hyperparameter optimization framework. The values of these parameters were chosen based on their performance during multiple rounds of training and validation. Based on our experiments, the optimal hyperparameters for TabTransformer are a model architecture with hidden layers of size 128, 2048, and 128, dropout probability of 0, “ELU” activation function, a learning rate of 0.00023, “AdamW” optimizer, weight decay of 0, and a batch size of 96.

### Transfer learning for case-control classification

To build models which take into account both senescent and clinical status, we employed a transfer learning strategy. Initially, we trained a model as a regressor to predict age in an age dataset. Subsequently, we froze the model weights, excluding the final layer, and re-trained the resulting model as a case-control classifier. The derived SHAP values indicate the significance of each molecular feature in disease development concerning aging. This approach enabled us to determine the relative importance of various molecular features in driving disease development within the aging context. The pipeline is depicted in Figure 1.

### Feature importance analysis

SHAP values, a mathematical method of feature importance analysis that constitutes a robust and interpretable technique for elucidating the contributions of individual features in complex predictive models, is based on the Shapley value from game theory and involves computing the contribution of each feature to explain the final predictions of machine learning models [24]. SHAP values explain individual predictions and identify the most important features in the model. Additionally, the employment of SHAP values for feature analysis offers a considerable advantage in multimodal settings, where discerning the interplay between various factors is indispensable for the development of accurate and efficacious aging clocks.

### Pathway enrichment analysis

Pathway enrichment analysis was performed on the list of top-100 genes ranked by their SHAP values obtained from the regressor model with the pathways available on the Reactome database. R package Enrichr was used to calculate the enrichment levels and *p*-values. Pathways with *p*-value < 0.05 were considered as significantly enriched.

### Manual analysis of resulting targets

Combination of aging clocks and target ID represents an interesting approach to identifying targets for aging-associated diseases. To illustrate the applicability of this approach for target identification, we have investigated 4 diseases associated with aging: idiopathic pulmonary fibrosis, chronic obstructive pulmonary disease (COPD), Parkinson’s disease (PD), and heart failure in PandaOmics. Gene lists of top-200 genes ranked by expression classifiers were used in Target ID projects for the mentioned diseases, along with a filter for small molecules to identify potential druggable proteins across these lists.

## Supplementary Materials

Supplementary Figures

Supplementary Tables 1-2

Supplementary Tables 3-8
