# Supplementary material for: Precious1GPT: multimodal transformer-based transfer learning for aging clock development and feature importance analysis for aging and age-related disease target discovery
Source: Aging (Albany NY). 2023 Jun 13;15(11):4649–66. doi: 10.18632/aging.204788 (PMC10292881; doi:10.18632/aging.204788)
Supplement: Supplementary Figures [file aging-15-204788-s001.pdf]

SUPPLEMENTARY FIGURES

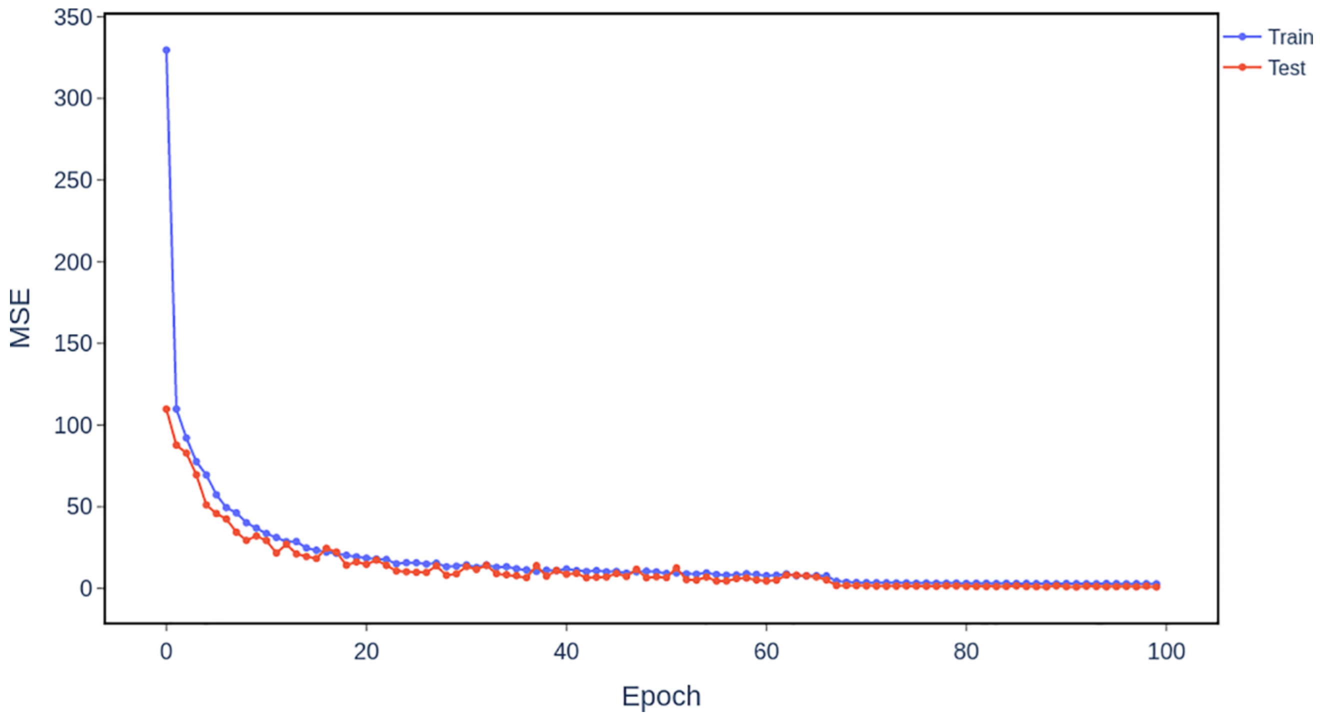

Supplementary Figure 1. Multimodal transformer learning curves on the train and 20% hold-out test datasets.

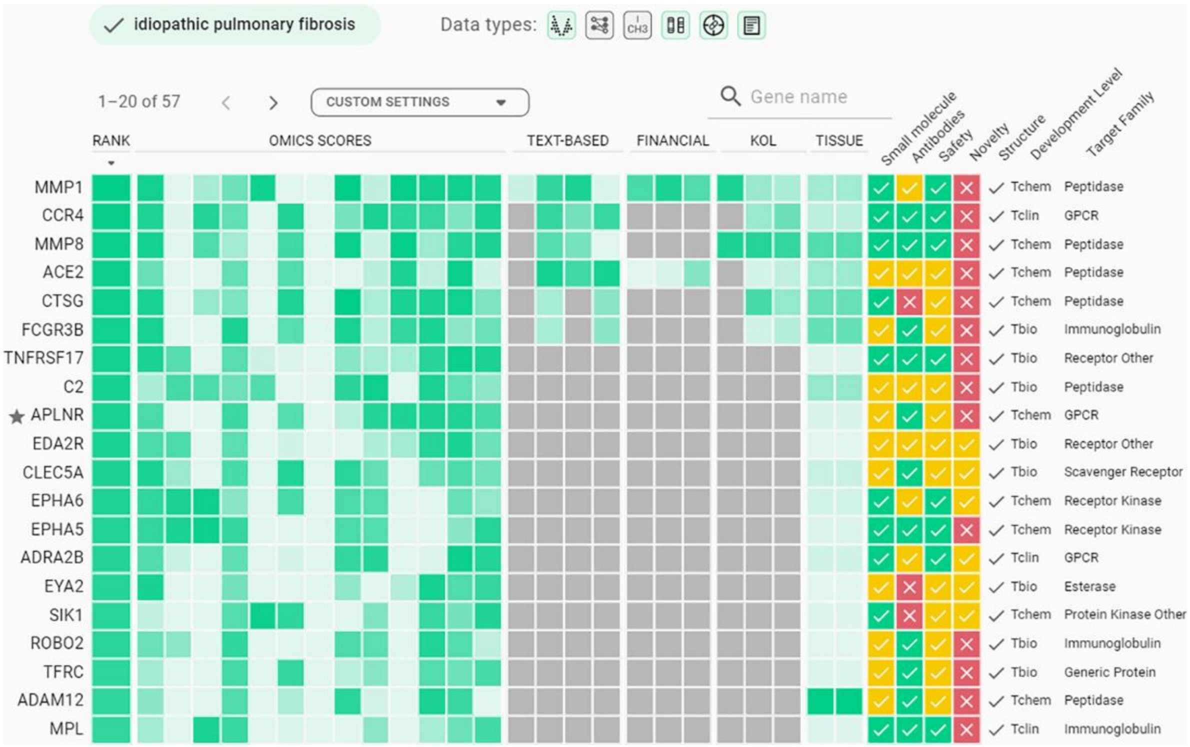

Supplementary Figure 2. Example of target ID output for idiopathic pulmonary fibrosis. Top-200 genes from expression classifiers were applied as a gene list in PandaOmics corresponding project for idiopathic pulmonary fibrosis, and a filter for small molecules was applied to identify druggable targets. Twenty genes highly ranked by PandaOmics are shown.

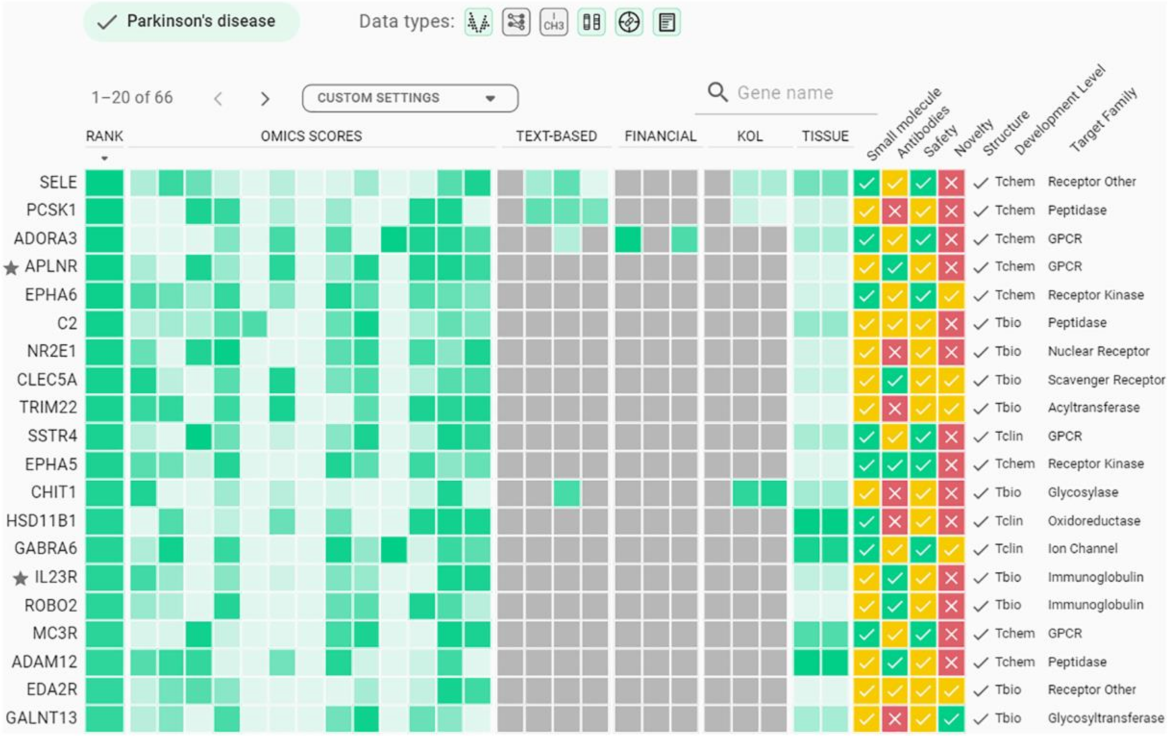

Supplementary Figure 3. Example of target ID output for Parkinson's disease. Top-200 genes from expression classifiers were applied as a gene list in PandaOmics corresponding project for PD, and a filter for small molecules was applied to identify druggable targets. Twenty genes highly ranked by PandaOmics are shown.

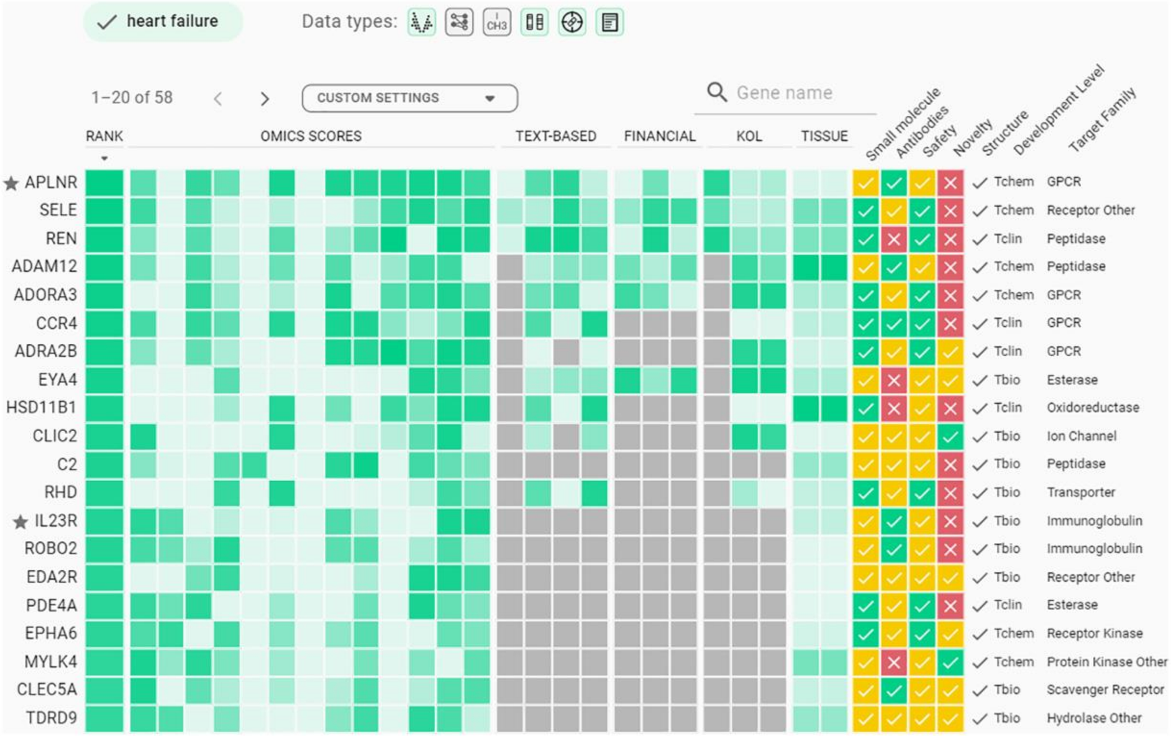

Supplementary Figure 4. Example of target ID output for heart failure. Top-200 genes from expression classifiers were applied as a gene list in PandaOmics corresponding project for heart failure, and a filter for small molecules was applied to identify druggable targets. Twenty genes highly ranked by PandaOmics are shown.

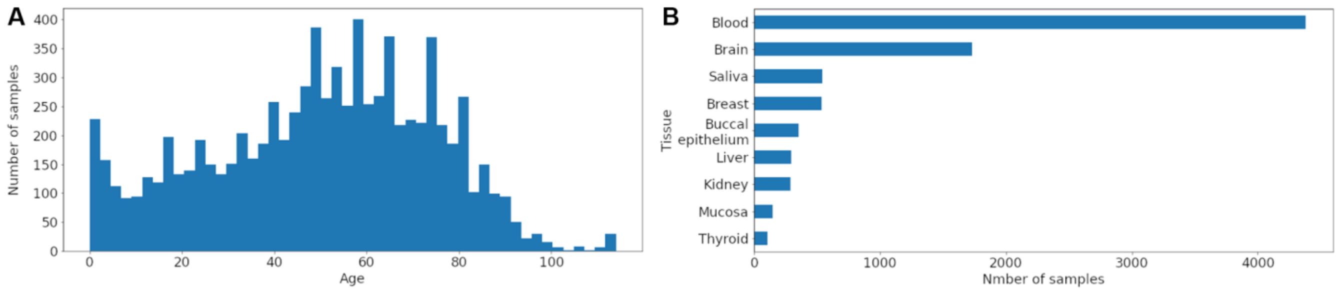

Supplementary Figure 5. Distribution by age (**A**) and tissues (**B**) for DNAm samples. Data was obtained from CNCB EWAS data hub. Ages distributed from 0 to 110 years. Most of the samples are blood samples.

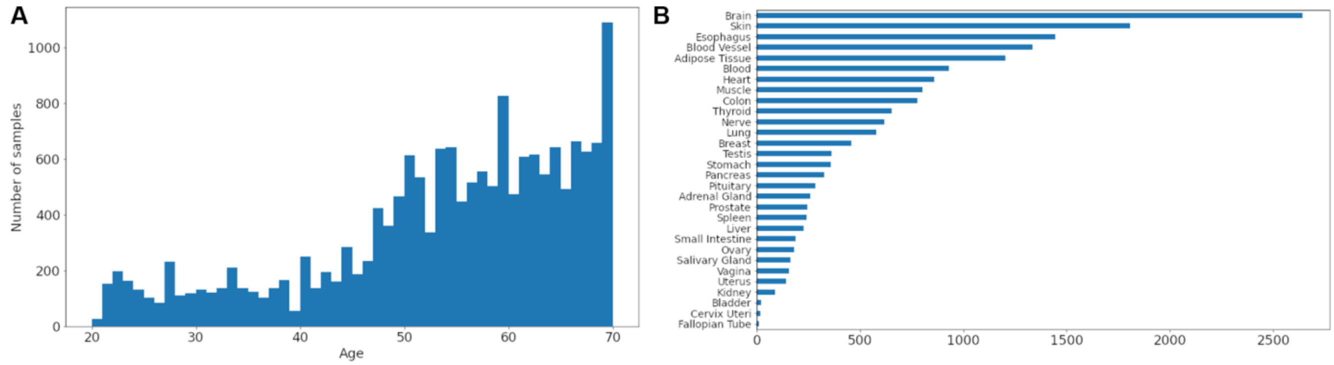

Supplementary Figure 6. Distribution by age (**A**) and tissues (**B**) for RNA-seq samples. Data are obtained from the GTEx project. Ages are distributed between 20 and 70 years. Brain and Skin samples comprise a bigger part of the dataset.
