# Supplementary material for: Precious1GPT: multimodal transformer-based transfer learning for aging clock development and feature importance analysis for aging and age-related disease target discovery
Source: Aging (Albany NY). 2023 Jun 13;15(11):4649–66. doi: 10.18632/aging.204788 (PMC10292881; doi:10.18632/aging.204788)
Supplement: Supplementary Tables 1-2 [file aging-15-204788-s002.pdf]

SUPPLEMENTARY TABLES

Supplementary Table 1. 5-fold cross-validation for multimodal transformer age prediction.

Metric	Combined	Methylation	Expression
MAE	5.800+/−0.437	4.815+/−0.458	6.469+/−0.427
RMSE	7.665+/−0.436	6.680+/−0.520	8.266+/−0.403
R2	0.823+/−0.021	0.923+/−0.013	0.572+/−0.041
MdAE	4.546+/−0.517	3.569+/−0.486	5.335+/−0.588

Supplementary Table 2. Performance of multimodal model on different combinations of tissues and data modalities. Estimates on 20% tissue-stratified hold-out test dataset.

TISSUE	MODALITY	MAE	R2	RMSE	MSE	MdAE	MAD	TEST_SAMPLES
Thyroid	METHYLATION	2.456	0.900	4.287	18.381	0.567	0.609	21
Buccal epithelium	METHYLATION	2.656	0.972	3.618	13.092	1.707	1.669	69
Saliva	METHYLATION	3.170	0.959	4.237	17.948	2.249	2.069	107
Mucosa	METHYLATION	3.358	0.896	3.988	15.903	2.879	3.010	29
Brain	METHYLATION	3.749	0.949	6.381	40.716	1.851	1.922	345
Blood	METHYLATION	4.291	0.928	5.990	35.880	3.168	3.177	863
Brain	EXPRESSION	4.483	0.640	5.954	35.446	3.539	3.372	528
Blood Vessel	EXPRESSION	5.229	0.715	6.680	44.621	4.159	4.515	267
Thyroid	EXPRESSION	5.367	0.698	6.967	48.539	4.406	4.195	129
Nerve	EXPRESSION	5.461	0.656	7.075	50.061	4.479	4.448	124
Testis	EXPRESSION	5.714	0.673	7.298	53.268	5.423	5.411	72
Breast	METHYLATION	5.797	0.738	7.738	59.876	4.477	4.443	106
Liver	METHYLATION	6.036	0.744	7.749	60.047	5.300	4.857	59
Ovary	EXPRESSION	6.070	0.672	8.488	72.052	4.460	4.425	36
Adrenal Gland	EXPRESSION	6.085	0.683	7.431	55.216	5.729	5.729	52
Pituitary	EXPRESSION	6.154	0.199	7.633	58.267	5.666	5.091	56
Small Intestine	EXPRESSION	6.204	0.708	7.533	56.739	6.014	6.007	38
Adipose Tissue	EXPRESSION	6.210	0.529	7.976	63.624	5.219	5.279	241
Salivary Gland	EXPRESSION	6.296	0.532	8.413	70.781	4.595	4.454	33
Kidney	METHYLATION	6.315	0.908	7.951	63.222	5.968	5.826	58
Skin	EXPRESSION	6.602	0.538	8.664	75.069	5.031	4.817	362
Esophagus	EXPRESSION	6.647	0.626	8.317	69.169	5.484	5.507	289
Prostate	EXPRESSION	6.670	0.669	8.349	69.711	5.096	5.345	49
Uterus	EXPRESSION	6.724	0.619	8.063	65.016	5.714	5.905	29
Lung	EXPRESSION	7.018	0.399	9.223	85.067	5.069	5.393	115
Breast	EXPRESSION	7.037	0.521	8.919	79.541	5.798	5.230	92
Muscle	EXPRESSION	7.040	0.558	8.604	74.027	5.981	5.584	160
Heart	EXPRESSION	7.059	0.403	8.799	77.421	6.302	6.405	172
Colon	EXPRESSION	7.204	0.590	9.143	83.598	6.241	6.118	156
Stomach	EXPRESSION	7.242	0.469	8.924	79.640	6.177	6.177	72
Pancreas	EXPRESSION	7.291	0.429	9.043	81.774	6.549	6.176	65
Vagina	EXPRESSION	7.436	0.311	9.372	87.826	5.883	5.364	31

Liver	EXPRESSION	7.592	0.243	9.372	87.829	7.066	5.343	45
Blood	EXPRESSION	8.158	0.397	10.617	112.724	6.288	6.303	186
Spleen	EXPRESSION	9.050	0.346	11.204	125.526	6.770	7.227	48
Kidney	EXPRESSION	9.715	−0.118	12.731	162.079	8.318	7.508	18
